# Delineation of Oral Mucosa as a Pseudo-Organ-at-Risk May Lead to a Decrease in the Incidence of Oral Mucositis: A Dosimetric Analysis of Intensity-Modulated Radiation Therapy Plans in Head and Neck Cancers

**DOI:** 10.7759/cureus.23716

**Published:** 2022-03-31

**Authors:** Himanshi Khattar, Piyush Kumar, Navitha S

**Affiliations:** 1 Department of Radiation Oncology, Shri Ram Murti Smarak Institute of Medical Sciences, Bareilly, IND

**Keywords:** pseudo-oar, head and neck cancers, mucositis, imrt, oral mucosa

## Abstract

Introduction

Oral mucositis is a common and potentially serious complication of radiation in head and neck cancer treatment. Severe mucositis causes pain, difficulty in chewing and swallowing that can cause treatment breaks and can cause treatment failures leading to local recurrence or a decrease in overall survival. The contouring of the planning target volume (PTV) and organ at risk (OAR) leaves some undefined regions on computed tomography imaging. The treatment planning system uses these regions as dumping sites for the dose in order to achieve the optimum plan. The present study was done to assess the dose going to these unspecified regions termed as pseudo-OARs and whether delineation of these structures and prescribing a dose constraint will reduce the dose to the oral mucosa without compromising the quality of the treatment plan.

Methods

Twenty patients of head and neck cancer were selected and were randomly placed in two arms. One was intensity-modulated radiation therapy (IMRT) arm I, which included the initial plans with which the patients were treated where the pseudo-OAR was not delineated and hence was not considered in the planning process. After giving treatment, the pseudo-OAR was delineated to see the dose dumped into the area outside the PTV. The other was IMRT arm II, where another virtual plan of the same patients of arm I was made wherein the delineation of the pseudo-OAR was done before planning and dose constraint prescribed. The pseudo-OAR consisted of anterior oral mucosa, part of mandible and maxilla, which was adjacent to the PTV in oropharynx and contralateral buccal mucosa patients. The dose constraint given to the pseudo-OAR was Dmean ≤30 Gy. Statistical significance was calculated by using a paired t-test. A p-value of <0.05 was considered as statistically significant.

Results

The dosimetric parameters of PTV were comparable in both the IMRT arms. The pre-specified objective was fulfilled with both the study arms. The dose homogeneity and conformity was also similar. The dosimetric parameters of other OARs were within the prescribed dose constraints. The Dmean value of the pseudo-OAR in arm I was 31.28 Gy ± 3.55 Gy and 7.87 Gy ± 9.11 Gy in arm II and the p-value was significant (p=0.001), whereas the Dmax in arm I was 61.82 Gy ± 5.91 Gy and 61.23 Gy ± 5.54 Gy in arm II (p=0.6). The dose to the pseudo-OAR in IMRT arm II was reduced drastically by 75%, which was statistically significant.

Conclusion

The delineation of oral mucosa as a pseudo-OAR should routinely be done. The dose constraints need to be optimized by clinical studies, which might probably decrease the incidence and severity of oral mucositis.

## Introduction

Oral mucositis is a common and potentially serious complication of radiation in head and neck cancer treatment. Severe mucositis causes pain, and difficulty in chewing and swallowing that can cause treatment breaks and can cause treatment failures leading to local recurrence or a decrease in overall survival [1,2].

Intensity-modulated radiation therapy (IMRT) has played a crucial role in enhancing outcomes and reducing morbidity in head and neck cancer patients. It utilizes the basic concept of the radiotherapy treatment where the maximum dose should be delivered to the tumor and minimum dose to the normal tissues [3]. To achieve this, different areas in the computed tomography (CT) scans of the patient are identified. The regions of the tumor along with the region of the potential microscopic spread of the tumor are identified as planning target volume (PTV). The various normal tissues, which may cause morbidities and are known as organs at risk (OARs), such as parotid glands (xerostomia), cochlea (hearing loss), spinal cord and brainstem (neurological morbidity), are also identified so that the dose restriction could be planned in these regions.

The IMRT planning uses many degrees of freedom to optimize a dose distribution according to the dose constraints prescribed. The contouring of the PTV and OAR leaves some undefined regions on CT imaging. The treatment planning system uses these regions as dumping sites for the dose in order to achieve the optimum plan [4]. In head and neck cancer patients, oral mucosa is not usually identified in the CT imaging that leads to high doses to these undefined regions in order to achieve optimum doses to the tumor and various OARs.

The present study was conducted to assess the dose going to these unspecified regions called pseudo-OARs in patients treated by IMRT plans and whether delineation of these structures and prescribing a dose constraint will reduce the dose to the oral mucosa without compromising the quality of the treatment plan.

## Materials and methods

Study population and radiotherapy planning

A total of 10 head and neck cancer patients (six carcinoma buccal mucosa cases and four carcinoma oropharynx) who had been treated by IMRT were selected for the retrospective study. All the patients underwent simulation in a supine position with neck rest and shoulder traction using a five-point thermoplastic cast. Contrast-enhanced CT scans of 3-mm slice thickness were obtained. The following volumes were delineated: gross tumor volume (GTV), which included the gross tumor visible clinically and radiologically in oropharynx cancer; clinical target volume primary (CTV-P), which included the post-operative tumor bed and entire primary site in the case of buccal mucosa while complete primary site in oropharynx cancer including GTV; CTV nodal (CTV-N), in which nodal volumes were delineated as per the guidelines given by Biau et al.; CTV final (CTV-final) including both CTV-P and CTV-N; and PTV, with a 5-mm isotropic margin to the CTV-final to account for setup errors [5].

OARs, which were delineated as per Danish Head and Neck Cancer Group (DAHANCA) guidelines, included spinal cord, mandible, parotids, lips, left and right cochlea, brainstem, left and right eye, left and right lens, and left and right optic nerve [6]. An isotropic expansion of 5 mm was given from the spinal cord for planning risk volume (PRV) spine and a 3-mm margin from brainstem was given for PRV brainstem.

Dose prescription and planning

A total dose of 60 Gy in 30 fractions was prescribed to the PTV in post-operative cases of buccal mucosa and 70 Gy in 35 fractions in oropharynx cases. All the patients were treated by IMRT. The constraints given for the OARs were as follows: PRV spine Dmax <50 Gy; mandible Dmax <70 Gy; lips Dmean <30 Gy; PRV brainstem Dmax <54 Gy; left and right cochlea Dmean <45 Gy; left and right parotid (combined) Dmean <26 Gy; left and right eye Dmax <50 Gy; left and right lens Dmax <7 Gy; left and right optic nerve Dmax <55 Gy.

All the IMRT plans were created with a 6 MV photon beam in Eclipse 13.6 treatment planning system (Varian Medical Systems, Palo Alto, CA). A total of nine coplanar beams with beam angles 0°, 40°, 80°, 120°, 160°, 200°, 240°, and 280° were used. An odd number of beams were used to avoid opposing beams. The isocentre was maintained at the same position in Phase I as well as Phase II to avoid the random setup error. Progressive resolution optimizer (PRO) was used in the optimization process with a grid size of 2.5 mm.

The OAR dose constraints and target dose parameters were followed as per the Radiation Therapy Oncology Group (RTOG) guidelines and evaluated as per International Commission on Radiation Units and Measurements (ICRU) 83 recommendations [7,8]. Objectives of OARs and target were maintained the same in both arms except for pseudo-OAR. A post-optimization AAA algorithm was used for dose calculation with the smart leaf motion calculator (LMC). Jaw tracking was also used to reduce multileaf collimator transmission. Cumulative dose volume histogram and isodose coverage were used for target and OAR dose evaluation.

IMRT Arm 1

This included the initial plans with which the patients were treated where the pseudo-OAR was not delineated and hence not considered in the planning process. After treatment, the pseudo-OAR was delineated to see the dose dumped into this area.

IMRT Arm 2

Here, another virtual plan was made of the same patients from arm 1 where delineation of the pseudo-OAR was done before planning and dose constraint prescribed.

**Figure 1:**
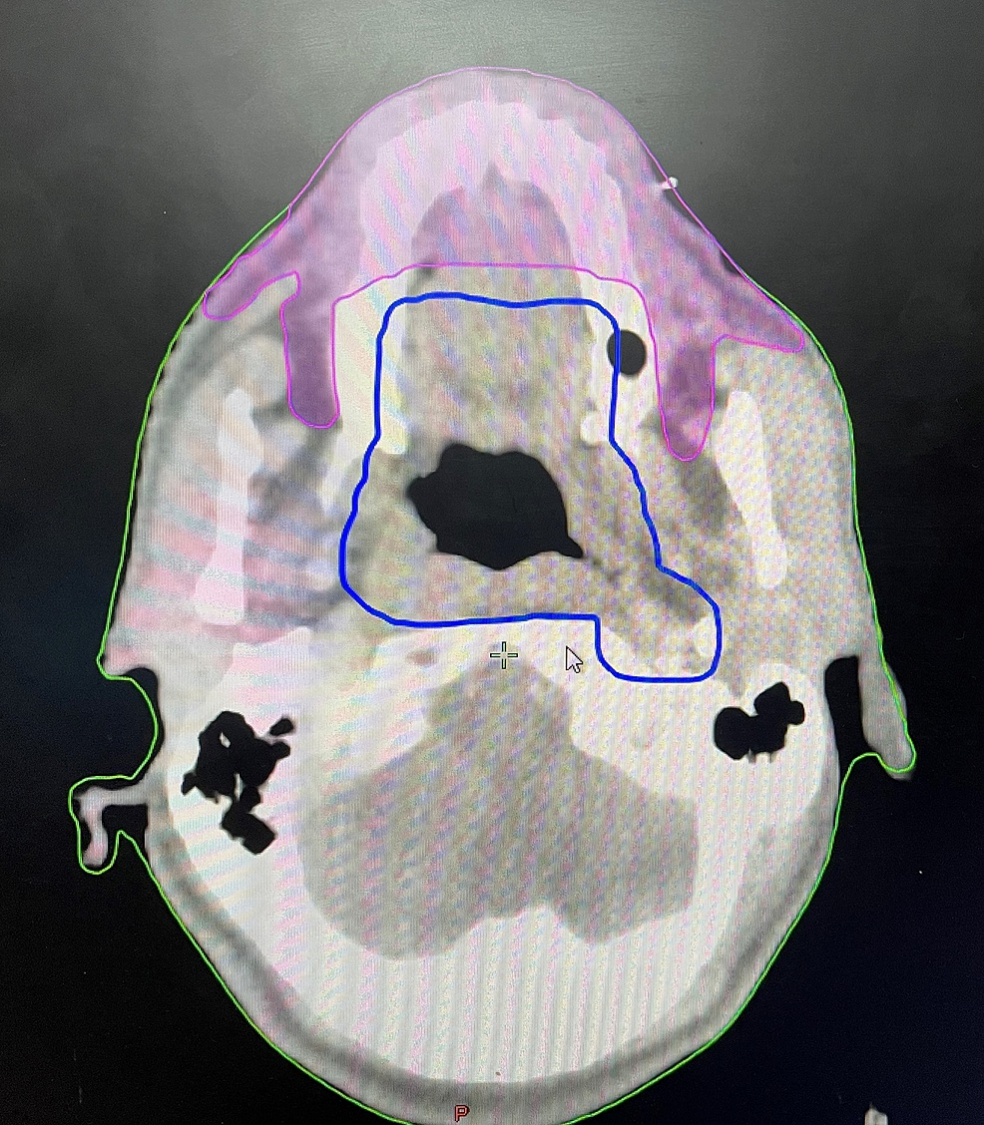
Cross-sectional CT scan image showing the delineation of PTV (blue) and pseudo-OAR (pink) CT, computed tomography; PTV, planning target volume; OAR, organ at risk

**Figure 2:**
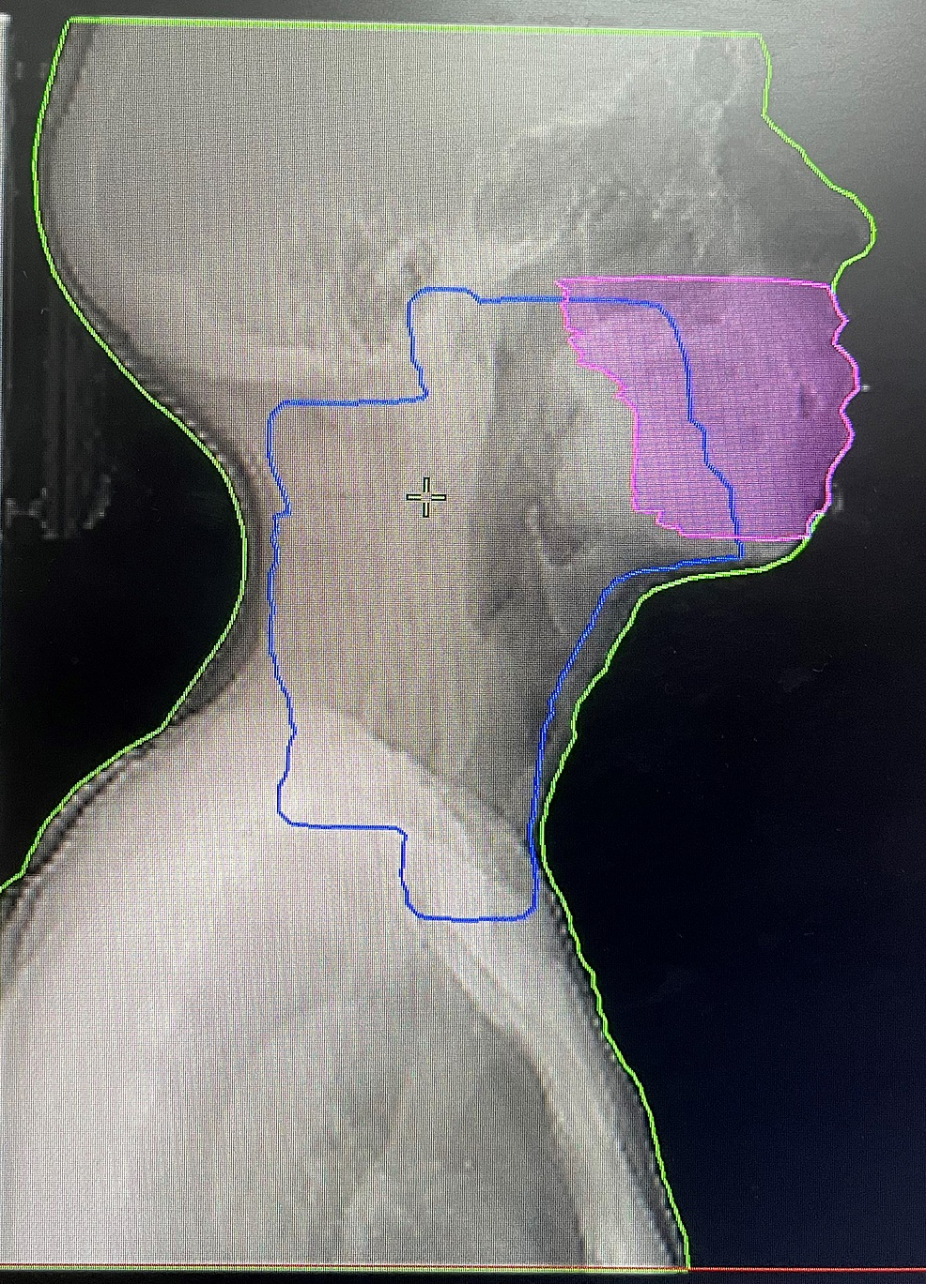
Digitally reconstructed radiograph in the lateral view shows PTV (blue) and pseudo-OAR (pink) PTV, planning target volume; OAR, organ at risk

The pseudo-OAR consisted of anterior oral mucosa, part of mandible and maxilla, which was adjacent to the PTV in oropharynx cancer patients, and contralateral buccal mucosa, part of maxilla and mandible, which was adjacent to the PTV in buccal mucosa patients (Figures 1, 2). The dose constraint given to the pseudo-OAR was Dmean ≤30 Gy.

Dosimetric assessment

Dosimetric parameters were assessed and compared in both the IMRT plans (IMRT arms 1 and 2). The dosimetric parameters for PTV were D95%, D50%, D2% (Dnear max), D98% (Dnear min), conformity index (CI) and homogeneity index (HI). OAR Dmax was calculated for spinal cord, brainstem, mandible ,eyes, lens, optic nerve, and optic chiasma, and Dmean was calculated for parotid, cochlea and lips. Doses to pseudo-OARs were compared in both IMRT arms 1 and 2. The IMRT plan was approved if 95% of the dose was received by 100% of PTV with CI <1.2 and HI <0.2.

Statistical analysis

Statistical significance was calculated by using a paired t-test. A p-value of <0.05 was considered as statistically significant.

## Results

The dosimetric parameters of PTV were comparable in both IMRT arms. The pre-specified objective was fulfilled with both the techniques with more than 95% of the volume receiving 95% of the prescribed dose. The dose homogeneity and conformity were also similar (Table 1).

**Table 1:** Dosimetric parameters of the planning target volume in two plans (mean±SD) IMRT, intensity-modulated radiation therapy; SD, standard deviation

Parameters	IMRT 1	IMRT 2	p-value
D95%	62.99±5.26	62.5±5.33	0.26
				D50%	63.34±6.51	64.95±4.88	0.4
D2% (Dnear max)	66.90±6.51	62.02±0.27	0.2				
				D98% (Dnear min)	61.07±15.46	66.48±4.96	0.18
Conformity index	1.16±0.22	1.13±0.05	0.69				
				Homogeneity index	0.83±0.06	0.07±0.04	0.5

The dose to pseudo-OARs in IMRT arm 2 was reduced drastically by 70%, which is statistically significant (Table 2).

**Table 2:** Dosimetric parameters of the pseudo-OAR in two IMRT plans (mean±SD) IMRT, intensity-modulated radiation therapy; SD, standard deviation; OAR, organ at risk

Pseudo-OAR	IMRT 1	IMRT 2	p-value
Dmean	31.28±3.55	24.54±9.11	<0.000001
Dmax	61.82±5.91	61.23±5.54	0.6

The dosimetric parameters of other OARs were comparable except in left cochlea (p=0.04), right optic nerve (p=0.01) and left eye (p=0.02) that were higher in IMRT arm 1, but these were within the prescribed dose constraints (Table 3).

**Table 3:** Dosimetric parameters of the OARs in two techniques (mean±SD) IMRT, intensity-modulated radiation therapy; SD, standard deviation; OAR, organ at risk

	IMRT 1	IMRT 2	p-value
Brainstem	33.40±14.73	35.96±11.24	0.64
Right optic nerve	5.57±5.14	4.86±4.49	0.01
Left optic nerve	4.88±4.12	4.36±3.76	0.11
Right eye	11.64±11.56	7.88±9.11	0.08
Left eye	7.39±8.33	6.19±7.42	0.02
Right lens	5.32±7.24	2.66±1.56	0.28
Left lens	3.13±2.01	2.77±1.86	0
Parotid right	40.19±17.33	41.47±16.97	0.65
Parotid left	44.30±12.38	43.75±12.27	0.74
Left cochlea	19.69±14.57	18.26±13.54	0.04
Right cochlea	22.83±11.11	22.11±11.49	0.44
Lips	26.05±5.01	24.18±7.00	0.11
PRV spine	42.46±4.45	42.25±4.38	0.64
Mandible	67.61±4.97	67.52±5.32	0.8

## Discussion

Radiotherapy of cancers of oropharynx and post-operatively buccal mucosa leads to oral mucositis that may cause pain and swallowing difficulties [9]. In cancers of the tongue, the whole oral cavity is treated where oral mucositis is severe, but in oropharynx and buccal mucosa treatments, the majority of the oral cavity region and maxilla can be spared since these are not the part of the PTV.

A virtual critical structure or pseudo-OAR is an arbitrary region of interest where we do not want the treatment planning system to dump the radiotherapy dose and generate hot spots leading to clinical side effects [4]. Oral mucositis can be prevented if the radiotherapy dose to this region is controlled. This can be achieved if we can delineate oral mucosa and identify it as a pseudo-OAR and prescribe a dose constraint.

Studies have different methods and terminologies to utilize this concept of pseudo-OAR. Rosenthal et al. mentioned these pseudo-OARs as ‘non-target structures’ and is one of the first studies to discuss about these pseudo-OARs [10]. The advent of IMRT led to the treatment of the head and neck tumors typically by nine field angles that forced the entry of beam paths through those non-target tissues that were not irradiated in conventional three-dimensional conformal radiation therapy (3DCRT). This led to radiation-induced toxicities like anterior oral cavity mucositis, posterior scalp toxicities, headaches, nausea and vomiting, which were not present in 3DCRT. They quantified the anterior mandible dose of >34 Gy to be associated with oral mucositis. A severe grade of oral mucositis may lead to oral ulcerations and feeding tubes, which is distressful for the patient. In our study, we found that it was important to identify these non-target structures and restrict doses so that the optimum advantage of IMRT was seen. Prescribing a mean dose constraint of less than 30 Gy in our IMRT arm 2 seems feasible as per the results of Rosenthal et al.

Dean et al. had a novel method for the delineation of oral mucosa called mucosal surface contour (MSC) [11]. The MSC was defined as a 3-mm-thick wall of tissue, based on the work by Ueno et al. measuring the oral mucosal thickness at multiple sites in five cadavers using a reamer method [12]. The surfaces included were buccal mucosa, buccal gingiva, gingiva proper, lingual gingiva, lingual frenulum, alveolar mucosa, labial mucosa, labial gingiva, labial frenulum, mucosal surface of the floor of the mouth, mucosal surface of the tongue anterior to the terminal sulcus, and the mucosal surface of the hard palate. In our study also, we have delineated the similar regions (termed pseudo-OARs) that were outside the PTV and were highly prone to oral reactions.

The pseudo-OARs were contoured partly based on the work by Hoebers et al. used the term ‘minor oral including sublingual salivary tissue (MOIST) target’ for the clinically useful structure that was intended to contain the majority of the minor salivary glands located within the mucosa covering the linings of the oral cavity and anterior oropharynx [13]. The delineation was done with an aim to include the minor salivary glands in the oral cavity and thus to reduce the xerostomia. On the contrary, in our study the pseudo-OARs mainly consisted of anterior oral mucosa, part of mandible and maxilla, which was adjacent to the PTV in oropharynx patients, and contralateral buccal mucosa, part of maxilla and mandible, which was adjacent to the PTV in buccal mucosa patients. Moreover, the present study aimed to reduce the dose to the oral cavity in view to decrease oral mucositis and not xerostomia.

Basu and Bhaskar in their study, related to OARs in head and neck cancers, contoured the extended oral cavity as a salivation-related structure that included the space posterior to the arch of the mandible and maxilla, posteriorly limited by the uvula, soft palate, and the base of tongue [14]. The delineation is almost similar to our study but the present study did the delineation for decreasing the oral mucositis while the study by Basu et al. specifies for decreasing xerostomia.

Musha et al. conducted a study to predict acute radiation mucositis (ARM) using an oral mucosal dose surface (OMDS) model in head and neck cancer patients treated by carbon ion radiotherapy [15]. The region of ARM correlated with the high dose region of the OMDS model. The study showed that the palate with a Dmax >43 Gy and the tongue with a Dmax >54.3 Gy were associated with higher risk of grade 2-3 oral mucositis. In our study, the Dmax of the pseudo-OAR was almost similar in both IMRT plans, which was around 61 Gy. The Dmean had a significant reduction in doses to the pseudo-OAR (31.28 Gy vs. 7.87 Gy). It may be noted that we cannot reduce the dose to the oral mucosa in cases of oral cavity carcinoma, especially in the subsite-tongue, because the PTV will overlap the major portions of the pseudo-OAR. Therefore, this concept of delineation of the pseudo-OAR (oral mucosa) will be useful in cases of carcinomas of the oropharynx, hypopharynx, larynx and buccal mucosa where contouring a pseudo-OAR will decrease the dose dumping in these areas, thereby decreasing the incidence and severity of oral mucositis. Musha et al. did the study on patients treated with carbon ion radiotherapy and we may presume that X-ray-based radiotherapy may also predict similar clinical side effects after we calculate the relative biological effectiveness.

Otter et al. evaluated the risk of grade 3 oral mucositis and pharyngeal dysphagia using the atlas of complication incidence method to generate constraints based on dose volume histograms and to identify dosimetric parameters to predict the risk of grade 3 oral mucositis and pharyngeal dysphagia [16]. The patients who did not meet all constraints had 64% of grade 3 oral mucositis while the patients who met all the dose constraints had 26% grade 3 oral mucositis. This was supported by multivariate analysis that revealed that the mean dose to the oral mucosa is the only significant predictor of grade 3 oral mucositis. By this hypothesis, in our study we found a significant decrease in the mean doses in pseudo-OARs in the IMRT arm 2 plans (7.87 Gy vs. 31.28 Gy) where pseudo-OARs were delineated before the treatment plan. This may support the hypothesis of Otter et al. to decrease the grade 3 oral mucositis if pseudo-OARs are delineated and optimum dose constraints are prescribed to such regions. The Dmax in our study was almost similar in both study arms that may not be a strong predictor of severe oral mucositis, but the Dmean as per Otter et al. may have significant clinical relevance. The contouring of this pseudo-OAR structure is routinely not practiced in head and neck cancers treated by IMRT. In the present study, it was seen that by achieving dose constraints, we were able to drastically decrease the dose to this pseudo-OAR region that may have a significant clinical advantage by decreasing the severity of oral mucositis without compromising the PTV dosimetric parameters.

## Conclusions

In patients of head and neck cancers treated by IMRT, oral mucosa is not routinely contoured, therefore, leading to dose and clinical manifestation of mucositis. This study highlights the importance of the delineation of oral mucosa as a pseudo-OAR in head and neck cancer patients undergoing radiotherapy. Dose constraints need to be optimized by clinical studies, which will probably decrease the incidence and severity of oral mucositis.
